# Human Neural Larva Migrans Caused by *Ophidascaris robertsi* Ascarid

**DOI:** 10.3201/eid2909.230351

**Published:** 2023-09

**Authors:** Mehrab E Hossain, Karina J. Kennedy, Heather L. Wilson, David Spratt, Anson Koehler, Robin B. Gasser, Jan Šlapeta, Carolyn A. Hawkins, Hari Priya Bandi, Sanjaya N. Senanayake

**Affiliations:** Canberra Health Services, Canberra, Australian Capital Territory, Australia (M. Hossain, K.J. Kennedy, H.L. Wilson, C.A. Hawkins, H. Bandi, S.N. Senanayake);; Australian National University, Canberra (K.J. Kennedy, C.A. Hawkins, H. Bandi, S.N. Senanayake);; Commonwealth Scientific and Industrial Research Organization Australian Capital Territory, Canberra (D. Spratt);; University of Melbourne, Melbourne, Victoria, Australia (A. Koehler, R.B. Gasser);; University of Sydney, Sydney, New South Wales, Australia (J. Šlapeta);; The University of Sydney Institute for Infectious Diseases, Sydney (J. Šlapeta)

**Keywords:** *Ophidascaris*, *Ophidascaris robertsi*, brain lesion, human, helminth, nematode, python, eosinophilia, parasites, zoonoses, Australia

## Abstract

We describe a case in Australia of human neural larva migrans caused by the ascarid *Ophidascaris robertsi*, for which Australian carpet pythons are definitive hosts. We made the diagnosis after a live nematode was removed from the brain of a 64-year-old woman who was immunosuppressed for a hypereosinophilic syndrome diagnosed 12 months earlier.

*Ophidascaris* species are nematodes exhibiting an indirect lifecycle; various genera of snakes across the Old and New Worlds are definitive hosts. *O. robertsi* nematodes are native to Australia, where the definitive hosts are carpet pythons (*Morelia spilota*). The adult nematodes inhabit the python’s esophagus and stomach and shed their eggs in its feces. Eggs are ingested by various small mammals, in which larvae establish, serving as intermediate hosts (*1*). Larvae migrate to thoracic and abdominal organs (*1*–*3*) where, particularly in marsupials, the third-stage larvae may reach a considerable length (7–8 cm), even in small hosts (*3*,*4*). The lifecycle concludes when pythons consume the infected intermediate hosts (*3*). Humans infected with *O. robertsi* larvae would be considered accidental hosts, although human infection with any *Ophidascaris* species has not previously been reported. We report a case of human neural larva migrans caused by *O. robertsi* infection.

## The Study

A 64-year-old woman from southeastern New South Wales, Australia, was admitted to a local hospital in late January 2021 after 3 weeks of abdominal pain and diarrhea, followed by dry cough and night sweats. She had a peripheral blood eosinophil count (PBEC) of 9.8 × 10^9^ cells/L (reference range <0.5 × 10^9^ cells/L), hemoglobin 99 g/L (reference range 115–165 g/L), platelets 617 × 10^9^ cells/L (reference range 150–400 × 10^9^ cells/L), and C-reactive protein (CRP) 102 mg/L (reference range <5 mg/L). Her medical history included diabetes mellitus, hypothyroidism, and depression. She was born in England and had traveled to South Africa, Asia, and Europe 20–30 years earlier. She was treated for community-acquired pneumonia with doxycycline and had not recovered fully. 

A computed tomography (CT) scan revealed multifocal pulmonary opacities with surrounding ground-glass changes, as well as hepatic and splenic lesions. Bronchoalveolar lavage revealed 30% eosinophils without evidence of malignancy or pathogenic microorganisms, including helminths. Serologic testing was negative for *Strongyloides*. Autoimmune disease screening results were negative. The patient’s diagnosis was eosinophilic pneumonia of unclear etiology; she began taking prednisolone (25 mg/d) with partial symptomatic improvement.

**Figure 1:**
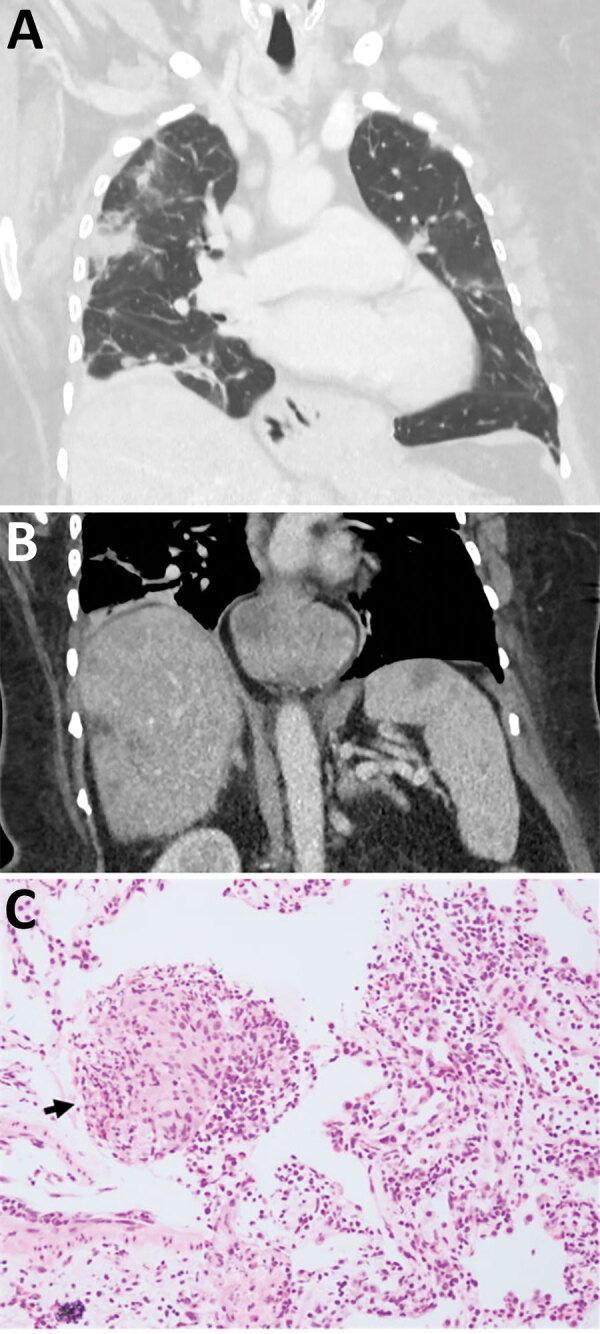
Early testing conducted during investigation of illness in a 64-year-old woman from southeastern New South Wales, Australia, who was later determined to have *Ophidascaris robertsi* nematode infection. A) Computed tomography scan of chest with venous contrast demonstrating multiple bilateral airspace opacities and nodules with a peripheral bronchovascular distribution. The opacities have surrounding ground-glass changes. Many were present in the patient’s study from a previous hospitalization; however, some had resolved while others were new, indicating a migratory pattern. B) Computed tomography scan of abdomen with venous contrast demonstrating multiple ill-defined hypoattenuated lesions within the liver and spleen. C) Hematoxylin and eosin stain (original magnification ×200) of a pulmonary lesion revealing prominent eosinophil infiltration of stroma and vessel walls. Arrow indicates a granuloma composed of histiocytes and eosinophils. The prominent eosinophilia was inconsistent with hypersensitivity pneumonitis, and the absence of vessel wall damage did not support a diagnosis of eosinophilic granulomatosis with polyangiitis.

Three weeks later, she was admitted to a tertiary hospital with recurrent fever and a persistent cough while on prednisolone. PBEC was 3.4 × 10^9^ cells/L and CRP was 68.2 mg/L. CT scans revealed persistent hepatic and splenic lesions and migratory pulmonary opacities (Figure 1, panels A, B). The pulmonary and hepatic lesions were 18F-fluorodeoxyglucose–avid on positive emission tomography scan. Lung biopsy specimen was consistent with eosinophilic pneumonia but not with eosinophilic granulomatosis with polyangiitis (EGPA) (Figure 1, panel C). Bacterial, fungal, and mycobacterial cultures were negative. *Echinococcus*, *Fasciola*, and *Schistosoma* antibodies were not detected; concentrated and fixed-stain techniques did not reveal parasites on fecal specimens.

We detected a monoclonal T-cell receptor gene rearrangement, suggesting T-cell driven hypereosinophilic syndrome (HES). Other hematologic and vasculitis investigations were unremarkable. HES treatment began with prednisolone (50 mg/d) and mycophenolate (1 g 2×/d). Because of her travel history, possibility of false-negative *Strongyloides* serology, and increased immunosuppression, she received ivermectin (200 µg/kg orally) for 2 consecutive days and a repeat dose after 14 days.

A CT scan in mid-2021 showed improvement in the pulmonary and hepatic lesions but unchanged splenic lesions. PBEC was 0.76 × 10^9^ in September 2021. We added mepolizumab (interleukin-5 monoclonal antibody, 300 mg every 4 wk) in January 2022 because we were unable to reduce the prednisolone below 20 mg daily without a flare of respiratory symptoms. When PBEC returned within normal range, we tapered the prednisolone dose.

**Figure 2:**
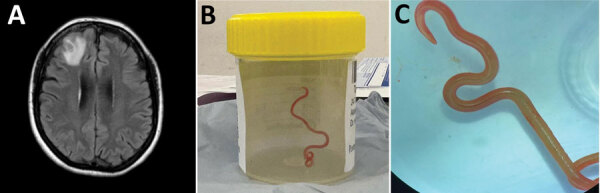
Detection of *Ophidascaris robertsi* nematode infection in a 64-year-old woman from southeastern New South Wales, Australia. A) Magnetic resonance image of patient’s brain by fluid-attenuated inversion recovery demonstrating an enhancing right frontal lobe lesion, 13 × 10 mm. B) Live third-stage larval form of *Ophidascaris robertsi* (80 mm long, 1 mm diameter) removed from the patient’s right frontal lobe. C) Live third-stage larval form of *O. robertsi* (80 mm long, 1 mm diameter) under stereomicroscope (original magnification ×10).

During a 3-month period in 2022, the patient experienced forgetfulness and worsening depression while continuing prednisolone (7.5 mg/d) and mycophenolate and mepolizumab at the same doses. PBEC was within reference range; CRP was 6.4 mg/L. Brain magnetic resonance imaging showed a 13 × 10 mm peripherally enhancing right frontal lobe lesion (Figure 2, panel A). In June 2022, she underwent an open biopsy. We noted a stringlike structure within the lesion, which we removed; it was a live and motile helminth (80 mm long, 1 mm diameter) (Figure 2, panels B, C). We performed a circumferential durotomy and corticotomy and found no other helminths. Histopathology of the dural tissue revealed a benign, organizing inflammatory cavity with prominent eosinophilia.

We provisionally identified the helminth as a third-stage larva of *Ophidascaris robertsi* on the basis of its distinctive red color, 3 active ascaridoid-like lips, presence of a cecum, and absence of a fully developed reproductive system, in the context of the known epidemiologic distribution of this species. The head and tail were preserved at the Australian National Wildlife Collection (W/LHC no. N5758). Small segments underwent independent PCR-based sequencing targeting the cytochrome oxidase c subunit 1 (*cox*1) (*5*,*6*) at the University of Sydney and the second internal transcribed spacer (ITS) 2 of nuclear ribosomal DNA (*7*) at the University of Melbourne. Both sequencing results provided >99.7% sequence match to *Ophidascaris* (formerly *Amplicecum*) *robertsi* isolates in the National Center for Biotechnology Information and in-house databases (Appendix).

A progress CT scan revealed resolution of pulmonary and hepatic lesions but unchanged splenic lesions. The patient received 2 days of ivermectin (200 µg/kg/d) and 4 weeks of albendazole (400 mg 2×/d). She was given a weaning course of dexamethasone (starting 4 mg 2×/d) over 10 weeks, while all other immunosuppression was discontinued. Six months after surgery (3 months after ceasing dexamethasone), the patient’s PBEC remained normal. Neuropsychiatric symptoms had improved but persisted.

## Conclusions

The patient in this case resided near a lake area inhabited by carpet pythons. Despite no direct snake contact, she often collected native vegetation, warrigal greens (*Tetragonia tetragonioides*), from around the lake to use in cooking. We hypothesized that she inadvertently consumed *O. robertsi* eggs either directly from the vegetation or indirectly by contamination of her hands or kitchen equipment.

The patient’s clinical and radiologic progression suggests a dynamic process of larval migration to multiple organs, accompanied by eosinophilia in blood and tissues, indicative of visceral larva migrans syndrome. We suspect that the splenic lesions are a separate pathology because they remained stable and were not PET avid, unlike the pulmonary and hepatic lesions. 

This case highlights the difficulty in obtaining a suitable specimen for parasitic diagnosis and the challenging management decisions regarding immunosuppression in the presence of potentially life-threatening HES. Although visceral involvement is common in animal hosts, the invasion of the brain by *Ophidascaris* larvae had not been reported previously. The patient’s immunosuppression may have enabled the larvae to migrate into the central nervous system (CNS). The growth of the third-stage larva in the human host is notable, given that previous experimental studies have not demonstrated larval development in domesticated animals, such as sheep, dogs, and cats, and have shown more restricted larval growth in birds and nonnative mammals than in native mammals (*4*).

After we removed the larva from her brain, the patient received anthelmintics and dexamethasone to address potential larvae in other organs. *Ophidascaris* larvae are known to survive for long periods in animal hosts; for example, laboratory rats have remained infected with third-stage larvae for >4 years (*4*). The rationale for ivermectin and albendazole was based on data from the treatment of nematode infections in snakes and humans (*8*,*9*). Albendazole has better penetration into the CNS than ivermectin (*10*). Dexamethasone has been used in other human nematode and tapeworm infections to avoid deleterious inflammatory CNS responses following treatment (*11*).

In summary, this case emphasizes the ongoing risk for zoonotic diseases as humans and animals interact closely. Although *O. robertsi* nematodes are endemic to Australia, other *Ophidascaris* species infect snakes elsewhere, indicating that additional human cases may emerge globally.

Appendix Additional information about human neural larva migrans with *Ophidascaris robertsi* ascarid.
