# Supplementary material for: Human Neural Larva Migrans Caused by Ophidascaris robertsi Ascarid
Source: Emerg Infect Dis. 2023 Sep;29(9):1900–3. doi: 10.3201/eid2909.230351 (PMC10461669; doi:10.3201/eid2909.230351)
Supplement: Appendix — Additional information about human neural larva migrans with Ophidascaris robertsi ascarid. [file 23-0351-Techapp-s1.pdf]

Article DOI: http://doi.org/10.3201/eid2909.230351

EID cannot ensure accessibility for supplementary materials supplied by authors. Readers who have difficulty accessing supplementary content should contact the authors for assistance.

Human Neural Larva Migrans Caused by *Ophidascaris robertsi* Ascarid

Appendix

Methods of Identification of the Nematode

At the University of Sydney, a small (≈3 mm) central section of the nematode (22P409183; W/LHC# N5758) was resected and used for DNA isolation (Monarch Genomic DNA Purification Kit, NEB, Australia). A fragment (≈600 base pair) of the cytochrome oxidase c subunit 1 (*cox*1) was amplified with LCO1490 (5′-GGT CAA CAA ATC ATA AAG ATA TTG G-3′) and HCO2198 (5′-TAA ACT TCA GGG TGA CCA AAA AAT CA-3′) primers (*1*). The PCR protocol used MyTaq Red Mix (Bioline) as previously described (*2*). The PCR product of the expected size was bidirectionally sequenced at Macrogen Inc. (Seoul, South Korea). Sequences were assembled and compared to available nematode sequences in CLC Main Workbench 22 (CLCbio, QIAGEN). Molecular *cox*1 identification confirmed that the nematode belonged to the genus *Ophidascaris*. Comparison with our in-house nematode *cox*1 sequence barcodes of *Ophidascaris* spp. from Australia, confirmed its close identity (99.7% across the amplified *cox*1 region) with a specimen from a carpet python (*Morelia spilota*) located in greater Sydney considered to represent *Ophidascaris robertsi*. The new *Ophidascaris robertsi cox*1 sequence is deposited in GenBank under the accession number OP198570. Its phylogenetic relationship to other available *Ophidascaris* species and related ascarid sequences is demonstrated in Appendix Figure 1.

At the University of Melbourne, genomic DNA was extracted from a small portion of the nematode larva by sodium dodecyl-sulfate:proteinase K treatment and purified over a spin column (Wizard Clean-Up, Promega). Subsequently, a region partially spanning the 5.8S and the second internal transcribed spacer of nuclear ribosomal DNA (ITS-2) was amplified from an aliquot of genomic DNA (5–10 ng) by PCR (*3*) using the oligonucleotide primers NC13: 5′-ATCGATGAAGAACGCAGC-3′ (forward) and NC2: 5′-TTAGTTTCTTTTCCTCCGCT-3′ (reverse), designed to regions of the 5.8S or 28S ribosomal RNA genes (*4*). PCR reactions

(50 μL) were performed in 10 mmol Tris–HCl, pH 8.4; 50 mmol KCl; 3.0 mmol MgCl2; 250 mmol each of dATP, dCTP, dGTP and dTTP; and 50 pmol of each primer with 1 unit Go*Taq* polymerase (Promega) under the following conditions: 94°C, 30 s (denaturation); 55°C, 30 s (annealing); 72°C, 30 s (extension) for thirty-five cycles, followed by a final extension at 72°C for five minutes (Thermocycler, Perkin Elmer Cetus). Positive (DNA from *Toxocara canis*; sample Tcan1) and negative (no-DNA) control samples were also included in the amplification run to exclude any 'carry-over' contamination. Following thermal cycling, aliquots (2 μL) of the test sample and the positive and negative control samples were examined on 1.5% agarose-TBE (65 mmol Tris–HCl, 27 mmol boric acid, 1 mmol EDTA, pH 9) gel, stained with ethidium bromide, and photographed upon transillumination. An aliquot (5 μL) of the amplicon (i.e., test sample) was treated with *Exo*SAP-IT (Affymetrix, USA), according to the manufacturer's instructions, and then subjected to direct, automated sequencing (BigDye Terminator v.3.1 chemistry, Applied Biosystems, USA) in both directions using the same primers (separately) as employed for PCR amplification. The ITS-2 sequence obtained by sequencing from the test sample-amplicon was then subjected to BLASTN analysis (via https://blast.ncbi.nlm.nih.gov/Blast.cgi) against all publicly available sequence datasets in the National Centre for Biotechnology Information (NCBI) database. The new *Ophidascaris robertsi* 5.8S to ITS-2 sequence is deposited in GenBank under the accession number OP886196. Its phylogenetic relationship to other available *Ophidascaris* species and related ascarid sequences is demonstrated in Appendix Figure 2.

References

1. Folmer O, Black M, Hoeh W, Lutz R, Vrijenhoek R. DNA primers for amplification of mitochondrial cytochrome c oxidase subunit I from diverse metazoan invertebrates. Mol Mar Biol Biotechnol. 1994;3:294–9. PubMed

2. Baron HR, Šlapeta J, Donahoe SL, Doneley R, Phalen DN. Compensatory gastric stretching following subtotal gastric resection due to gastric adenocarcinoma in a diamond python (*Morelia spilota spilota*). Aust Vet J. 2018;96:481–6. PubMed https://doi.org/10.1111/avj.12764

3. Mullis K, Faloona F, Scharf S, Saiki R, Horn G, Erlich H. Specific enzymatic amplification of DNA in vitro: the polymerase chain reaction. Cold Spring Harb Symp Quant Biol. 1986;51:263–73. PubMed https://doi.org/10.1101/SQB.1986.051.01.032

4. Chilton NB. The use of nuclear ribosomal DNA markers for the identification of bursate nematodes (order Strongylida) and for the diagnosis of infections. Anim Health Res Rev. 2004;5:173–87. PubMed https://doi.org/10.1079/AHR200497

5. Katoh K, Standley DM. MAFFT multiple sequence alignment software version 7: improvements in performance and usability. Mol Biol Evol. 2013;30:772–80. PubMed https://doi.org/10.1093/molbev/mst010

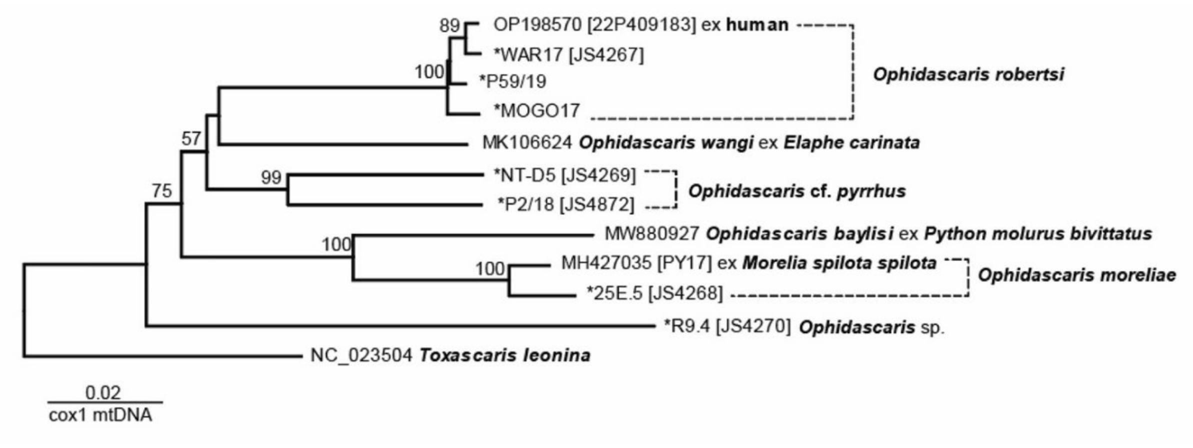

Appendix Figure 1. Phylogenetic relationship of *Ophidascaris robertsi cox*1. The tree was inferred using the minimum-evolution method and evolutionary distances were computed using the maximum composite likelihood method and are in the units of the number of base substitutions per site. The tree is drawn to scale; branch lengths are in the same units as those of the evolutionary distances used to infer the phylogenetic tree. The *cox*1 sequence alignment totals 1,470 positions. The percentage (>50%) of replicate trees in which the associated taxa clustered together in the bootstrap test (500 replicates) are shown next to the branches. Evolutionary analyses were conducted in MEGA11 (https://www.megasoftware.net). Sequences with * are unpublished, from a local in-house database at the University of Sydney, Sydney, Australia.

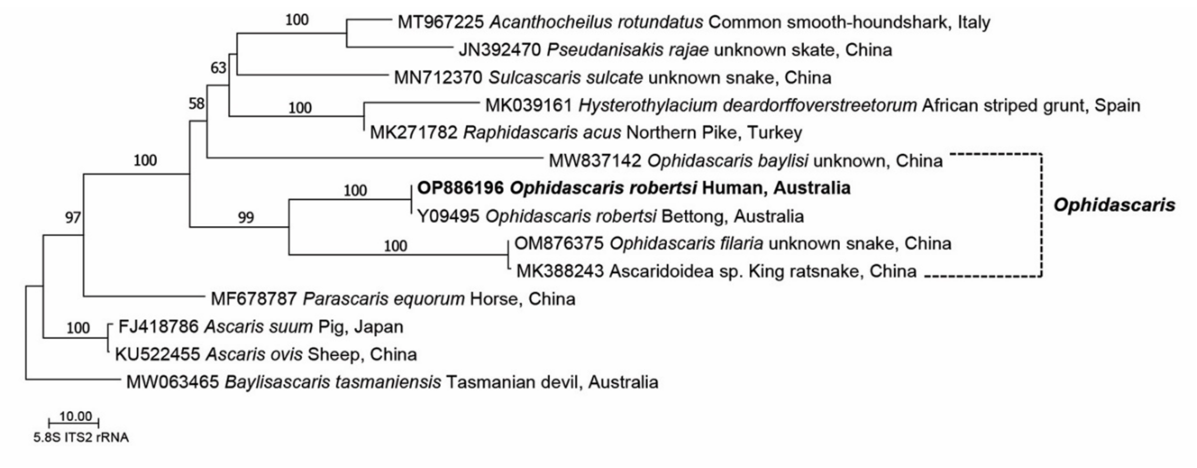

Appendix Figure 2. Phylogenetic relationship of *Ophidascaris robertsi* partial 5.8S and ITS-2 region of rRNA. The tree was inferred using the minimum evolution method and evolutionary distances were computed using the maximum composite likelihood method and are in the units of the number of base substitutions per site. The tree is drawn to scale; branch lengths are in the same units as those of the evolutionary distances used to infer the phylogenetic tree. The partial 5.8S and complete ITS-2 region of rRNA sequence alignment totals 592 positions and was aligned using MAFFT (*5*). The percentage (>50%) of replicate trees in which the associated taxa clustered together in the bootstrap test (2,000 replicates) are shown next to the branches. Evolutionary analyses were conducted in MEGA11 (https://www.megasoftware.net).
